# Sanitation and Hygiene Practices in Small Towns in Tanzania: The Case of Babati District, Manyara Region

**DOI:** 10.4269/ajtmh.19-0551

**Published:** 2020-08-17

**Authors:** Hoyce Mshida, Gabriel Malima, Revocatus Machunda, Alfred N. N. Muzuka, Joseph Banzi, Om Prasad Gautam, Mbaye Mbeguere, Kyla Smith, Sandy Cairncross, Edward S. Shana, Amadeus Herman, Karoli N. Njau

**Affiliations:** 1 Nelson Mandela African Institution of Science and Technology, Arusha, Tanzania;; 2 WaterAid Tanzania, Tanzania;; 3 WaterAid UK, London, United Kingdom;; 4 London School of Hygiene and Tropical Medicine, London, United Kingdom;; 5 Babati Water and Sanitation Authority, Babati, Tanzania;; 6 Babati Town Council, Babati, Tanzania

## Abstract

Formative research findings from the fast-growing Babati town were used to assess the prevalence of sanitation and hygiene practices among individuals and institutions and associated factors. A cross-sectional study involving household surveys, spot-checks, focus group discussions, in-depth interviews, and structured observations of behaviors showed that 90% of households have sanitation facilities, but 68% have safely managed sanitation services. The most common types of household sanitation facilities were pit latrines with slab (42%) followed by flush/pour flush toilets (32%). Therefore, the management of wastewater depends entirely on onsite sanitation systems. The majority of households (70%) do not practice proper hygiene behaviors. Thirteen percent of the households had handwashing stations with soap and water, handwashing practice being more common to women (38%) than men (18%). The reported handwashing practices during the four critical moments (handwashing with soap before eating and feeding, after defecation, after cleaning child’s bottom, and after touching any dirt/dust) differed from the actual/observed practices. Households connected to the town’s piped water supply were more likely to practice handwashing than those not directly connected. Sanitation and hygiene behaviors of the people in the study area were seen to be influenced by sociodemographic, cultural, and economic factors. The conditions of sanitation and hygiene facilities in public places were unsatisfactory. There is an urgent need to ensure that the sanitation and hygiene services and behaviors along the value chain (from waste production/source to disposal/end point) are improved both at the household level and in public places through improved sanitation services and the promotion of effective hygiene behavior change programs integrated into ongoing government programs and planning.

## INTRODUCTION

Poor access to sanitation and hygiene services is fast-growing problems in urban populations, particularly in least developed countries. The problems are acute especially in small growing towns where availability of water and sanitation facilities is inadequate.^1^ Use of toilets, the control of pollution and diseases related to fecal contamination of water sources, is a pressing issue. More than 2 billion people globally do not have access to even basic sanitation, and 673 million people are defecating in the open environment.^2^

Handwashing after defecation is one of the key components of hygiene behavior. However, only 19% of people globally are washing their hands with soap after defecation.^3^ Compounding the problem of poor access to sanitation and hygiene services are fast-growing urban populations. The world’s urban population is estimated to increase to 66% by 2050, which is from 3.9 billion in 2014 to 6.3 billion in 2050.^4^ Africa as a continent is projected to have one of the highest urban growth rates, mostly occurring in small towns.

As one of the Global South countries, Tanzania is facing similar challenges. According to a report from the WHO/UNICEF and Joint Monitoring Programme for Water Supply, Sanitation and Hygiene of 2018/2019, only 47% of Tanzanians have access to basic sanitation and 23.5% have access to basic hygiene facilities (handwashing with soap and water), and the increase of unplanned settlements has intensified the challenge of increasing access to environmental sanitation and hygiene services to urban dwellers.^5^ Poor sanitation costs Tanzania about 206 million United States Dollar (USD) annually; this includes the amount lost annually because of premature deaths caused by diarrhea, mostly contributed by poor water, sanitation, and hygiene; money spent each year on health care; productivity losses while sick or accessing health care; and cost of finding a private location to defecate.^6^ Less than 25% of the total population (urban and rural) has a designated place for handwashing with soap.^7^ Furthermore, only 38% of schools in Tanzania have an adequate number of toilets, 20% of schools have water supply facilities within the school premises, and less than 10% of all schools in the country have functioning handwashing facilities with available soap and water to enable children to maintain their personal hygiene and internalize relevant sanitation practices.^8^

Fast-growing towns do not have enough infrastructures in place to facilitate sanitation and hygiene services and Babati town, northern Tanzania, is no exception. The town’s population grew from 31,077 people in 2002 to 93,108 in 2012 and continued to grow to 110,000 in 2016.^9^ At the time of this study, not much was known about the level, status, and practice of the sanitation and hygiene. Thus, a formative research and baseline were conducted as part of a larger research project entitled “*Achieving universal access to adequate, sustainable and equitable sanitation and hygiene services in the Cities of Tomorrow*.” The project’s aims were to assess the current status of sanitation and hygiene practices in Babati town and its associated factors and inform the participatory planning process to better design the improvement plan. The research also aimed at contributing to the understanding of how sustainable universal sanitation and hygiene services can be provided in small towns or fast-growing urban centers.

## METHODS

### Study site.

Formative research was conducted in Babati town, from November 2016 to June 2017.^9^ It is a fast-growing town situated within the East African Rift Valley with an estimated population of 110,000 that lives within an area of 460.86 km^2^. Babati town consists of eight wards, and it was recently upgraded to regional headquarters of the Manyara region. Economic activities of Babati are primarily agriculture and small business.

### Study design.

A cross-sectional study design using qualitative and quantitative methods was applied for the research. Quantitative data were collected using a guided questionnaire (*n* = 486 households and *n* = 52 institutions), spot-check (*n* = 486), and structured observation to assess actual behaviors (*n* = 245) and to quantify the magnitude of the problems. The study assessed the sociodemographic, cultural, and economic profile, and existing water, sanitation, and hygiene status and practices. Qualitative data were collected using focus group discussions (FGDs), key informant interviews, and a disability audit (Table 1).

**Table 1:** Data collection techniques, size, and tools

Formative research (FR) method/studies	Sample size	Data collection tools	Analysis
Household survey	486 Households	Survey questionnaire	R program, SPSS
Spot-check – households	486 Households	Checklist	SPSS
Spot-check – institutions	31 Schools, two colleges, three market places, 14 restaurants/lodges	Checklist	SPSS
Focus group discussion	33 Sessions in schools (396 respondents), 16 sessions in communities	Guiding checklist	NVivo
Household survey	486 Households	Survey questionnaire	R program, SPSS
Spot-check – households	486 Households	Checklist	R program
Spot-check – institutions	31 Schools, two colleges, five HF, three market places, 14 restaurants/lodges	Checklist	R program
Structured observation of hygiene behaviors	245 Households	Structured observation checklist	R program
Structured observation of hygiene behaviors	Public toilets (one bus station, two markets)	Structured observation checklist	SPSS

The primary unit of sampling in this study was a household. The primary respondent for the household survey was the head of the household; however, when the head of household was not available for interview, another adult member of the household was interviewed. In the primary and secondary school and college settings, students and teachers were invited as participants. The head of organizations, local government authority personnel, heads of community networks and committees, sanitation entrepreneurs, and relevant focal points for sanitation and hygiene were key respondents from public and private institutions.

Spot-check observations were conducted in 486 households, 31 schools, one bus station, and three market places using a standard checklist to assess the availability of sanitation and handwashing facilities, their current condition, and functionality. Disability audits were conducted at the same time as the household survey to assess the current state of accessibility and usability of facilities by people with special needs. The disability audit checklist was adopted from WaterAid^10^ and United Republic of Tanzania.^11^

Structured observation was carried out in 245 randomly selected households which had a caregiver of at least one child younger than 5 years to assess current hygiene practices. Participants were told that the observation was to assess their daily routines to avoid any interference and observer’s effect into the behaviors. Observations were carried out in the morning (6:00–10:00 am) to observe the key behaviors of interest such as handwashing with soap at different times, food hygiene, and water-related behaviors as well as the cleanliness of the household environment. Observers were told to minimize making conversation during the observation to avoid unintended bias. Furthermore, the hygiene situation in public toilets at one bus station and one market was observed. The hygiene behaviors of customers attending these toilets were observed by trained enumerators from 06:00 to 18:00. A checklist was used to note down key hygiene behaviors practiced by the customers. The observation exercise was carried out by enumerators working in shifts over three consecutive days.

A total of 49 FGD sessions were conducted: 16 FGD sessions in the city wards involving household adult members and 33 FGDs in schools involving students from class five to seven and in colleges. Focus group discussion sessions were conducted to assess the school Water, Sanitation and Hygiene (WASH) status and menstrual hygiene management (MHM). Each session involved 12 participants. In each session, the participants were grouped by gender. The participants for the MHM FGDs were purposively selected among the girls at menarche. Selection of girls at menarche was made by the matron of the respective school. Furthermore, two specific FGDs were conducted involving students with psychosocial disabilities and hearing impairments. The two schools where these FGDs were conducted had special classes for students with psychosocial disabilities and hearing impairments, and therefore, the number of the students was large enough (*n* = 9) to form a group for FGDs. Students with physical impairments were also involved in the FGDs in some of the schools.

The study received ethical clearance from the National Institute for Medical Research, Tanzania. In addition, written consent was obtained from the study participants before they participated in the study. The study participants filled and signed the consent forms voluntarily.

### Data collection and quality check.

Before the commencement of the research, enumerators and facilitators of FGDs were trained (2- and 3-day trainings, respectively). The questionnaires and FGD guides were translated from English to Kiswahili. Pretesting of the data collection tools (3 days) was carried out outside of Babati town. At the end of each data collection day, the completed questionnaires were reviewed and cleaned by the numerators together with the supervisors. The FGDs were conducted in Kiswahili, recorded (with consent), and lasted approximately 60 minutes. The interview settings were selected to provide a comfortable environment for the interviewees to express themselves freely. The recruited participants for each FGD session were individuals who had resided in the particular ward/village for more than a year and came from different groups including socially marginalized groups (i.e., women, people with disability, and low-income earners). In addition, each group included at least one person with a disability, a farmer or livestock keeper, formal occupation, and a person older than 69 years.

### Data analysis.

#### Qualitative data analysis

The qualitative data analysis was conducted with the help of qualitative data analysis software, NVivo version 11 (QSR International, Burlington, MA). This software was used to organize and analyze unstructured data. The voice records were transcribed and translated into English and fed into the software, sorted, and coded. The generated coded information was then summarized according to main and subthemes.

#### Quantitative data analysis

Quantitative data were entered and analyzed using SPSS version 25 (International Business Machines Corporation [IBM], New York, NY). Initial dummy tables were produced to check the data errors and to perform a normality check. Descriptive analysis was then carried out to show the current status and practices of hygiene and sanitation. The univariate descriptive analysis generated the frequencies and percentages. In addition, the survey data were subjected to the chi-square test to test the associations of the variables. The chi-square test analyzed the association between the demographic and socialeconomic variables and type of sanitation facility the household owns or has access to. The variables considered in the chi-square tests were household head, education level, marital status, wealth quintile, residence ownership, source of water, family size, and household income.

## RESULTS

### Sociodemographic characteristics of the study participants.

The household survey showed that 63% and 37% of respondents were female and male, respectively, most of the participants being either married (74%) or widowed/divorced (16%). The age of respondents ranged from 18 to 75 years, and the majority (24%) were aged 31–40 years. The report from survey further showed that 78.8% of households were male-headed households. The majority of the respondents were living in a privately owned house (85%), the average family size was five, and 50% of the households had children younger than 5 years. More than half (57%) of respondents were engaged in crop farming as a primary occupation and livestock keeping as a secondary occupation. Sixty percent of the respondents had attained primary education, and the mean average monthly income of the respondents was 9 USD. The majority of the households surveyed had a family size of 5–8 individuals (56%) (Supplemental Table 1).

### Prevailing sanitation conditions in Babati town.

During FGDs, respondents discussed the sanitation status in their areas. It was revealed that most of the households in the study area use pit latrines, with mud superstructures and unroofed. One respondent explained, “*Our latrines are not in a good condition…... they are pit latrines, un-roofed…they don’t have permanent doors…therefore; sanitation to our community is not that good.*” In many FGDs, respondents said that their latrines are not improved because majority cannot afford to either install the improved ones because of lack of financial capacity to buy materials for constructing improved/modern toilets. One respondent argued, “*Many people fail to use improved latrines due to economic reasons. I like to install improved latrine, but my economic situation is not good, I have no other choice, I keep on using a (traditional) pit latrine.*” In addition, lack of water in some places was also mentioned as another reason for not installing improved latrines, *“we like to use improved latrines which are durable but* that is *where problem arises, some of us are living where water is not close to our homes, walk a long distance to get water.”*

Results from 486 households surveyed showed that 90% of the households own toilets, 3.3% do not, and 7.4% share their facilities with their neighbors. The most common type of household sanitation facility used was pit latrine with slab (42%), followed by flush or pour flush toilet (32%). Most facilities (81%) were situated inside the house or within 10 m from the house, and the majority (49%) of the households do not empty their toilets. When a latrine is full, it is abandoned, and a new one built. Furthermore, 35% responded that they have never have needed to empty their toilets as the pit has yet to fill (Supplemental Table 2). The sanitation facilities were further classified as per joint monitoring program (JMP) sanitation ladder (Table 2), where 68% of the facilities were found to be safely managed, although the rest could not.

**Table 2:** Sanitation classification according to JMP sanitation ladder

	*n*	%
Safely managed sanitation services*	330	67.9
Basic sanitation services†	16	3.3
Limited sanitation services‡	31	6.4
Unimproved sanitation services§	93	19.1
Open defecation‖	16	3.3
Total	486	100

* Use of improved facilities which are not shared with other households and where excreta are safely disposed in situ or transported and treated off-site.

† Use of improved facilities which are not shared with other households.

‡ Use of improved facilities shared between two or more households.

§ Use of pit latrines without a slab or platform, hanging latrines, or bucket latrines.

‖ Disposal of human feces in fields, forests, bushes, open bodies of water, beaches, and other open spaces or with solid waste.

Chi-square test results revealed that among the eight variables that were used to determine the association between the type of household sanitation facility and household social economic characteristics, four variables were significant. The significant variables were education level (χ^2^ = 16, *P* = 0.04), wealth quintile (χ^2^ = 21, *P* = 0.006), residence ownership (χ^2^ = 143, *P* < 0.001), and source of water for domestic use (χ^2^ = 72, *P* < 0.001). The type of sanitation facility a household owns also varies according to whether the house is privately owned or rented. Other variables, including head of the household, marital status, family size, and household income, did not have any association with the type of sanitation facility the household has (Table 3).

**Table 3:** Variables associated with types of toilets

		Safely managed		Basic		Limited		Unimproved		Open defecation		*P*-value
		*n*	*f* (%)	*n*	*f* (%)	*n*	*f* (%)	*n*	*f* (%)	*n*	*f* (%)	
Head of household	Male-headed household	263	68.7	12	3.1	23	6	73	19.1	12	3.1	0.934 (χ^2^ = 0.835)
	Female-headed household	67	65	4	3.9	8	7.8	20	19.4	4	3.9	
Education level	No formal education	32	69.6	1	2.2	0	0	10	21.7	3	6.5	0.04 (χ^2^ = 16.204)
	Primary education	196	67.8	6	2.1	24	8.3	53	18.3	10	3.5	
	Secondary education and above	48	80	4	6.7	3	5	5	8.3	0	0	
Marital status	Single	35	72.9	2	4.2	4	8.3	7	14.6	0	0	0.061 (χ^2^ = 20.31)
	Married/cohabiting	248	69.3	11	3.1	20	5.6	70	19.6	9	2.5	
	Divorced/separated	16	48.5	2	6.1	5	15.2	6	18.2	4	12.1	
	Widow	31	66	1	2.1	2	4.3	10	21.3	3	6.4	
Wealth quantile	High	250	72.3	7	2	26	7.5	52	15	11	3.2	0.006 (χ^2^ = 21.43)
	Second/third	3	50	0	0	0	0	3	50	0	0	
	Fourth	24	53.3	4	8.9	1	2.2	14	31.1	2	4.4	
Residence ownership	Privately owned house	287	69.5	13	3.1	7	1.7	91	22	15	3.6	0.00 (χ^2^ = 143.31)
	Relative house	16	84.2	1	5.3	1	5.3	0	–	1	5.3	
	Rental house	27	50	2	3.7	23	42.6	2	3.7	0	0	
Source of water	Borehole	20	54.1	0	0	1	2.7	14	37.8	2	5.4	0.00 (χ^2^ = 71.86)
	Buy from vendors	20	60.6	0	0	1	3	12	36.4	0	0	
	River/canal/spring	28	57.1	1	2	0	0	16	32.7	4	8.2	
	Other sources	136	68.3	5	2.5	7	3.5	43	21.6	8	4	
	Connected to the water supply system	126	75	10	6	22	13.1	8	4.8	2	1.2	
Family size	1–4	105	60.7	4	2.3	17	9.8	39	22.5	8	4.6	0.06 (χ^2^ = 14.94)
	5–8	192	70.1	11	4.0	14	5.1	49	17.9	8	2.9	
	9 and above	33	84.6	1	2.6	0	–	5	12.8	0	–	
Income (USD)	49 and below	109	63.4	3	1.7	10	5.8	43	25	7	4.1	0.44 (χ^2^ = 16.18)
	50–99	77	66.4	5	4.3	10	8.6	20	17.2	4	3.4	
	100–149	34	70.8	2	4.2	3	6.3	8	16.7	1	2.1	
	150–199	16	84.2	0	–	1	5.3	2	10.5	0	0	
	200 and above	34	82.9	2	4.9	1	2.4	4	9.8	0	0	

### Prevailing status of key hygiene behaviors.

The formative research assessed five key behaviors including 1) handwashing with soap at critical moments such as after defecation and before feeding/eating, 2) food hygiene, 3) water-related behaviors, 4) sanitation-related behaviors including child feces disposal, and 5) MHM (Table 4).

**Table 4:** Reported and observed key hygiene behaviors at household level

Hygiene behavior	Reported				Observed	
	Knowledge		Reported practices		Observed practice	
	Yes, *n* (%)	No, *n* (%)	Yes, *n* (%)	No, *n* (%)	Yes, *n* (%)	No, *n* (%)
Handwashing with soap (*N* = 468)					*N* = 195	
Handwashing facility present	NA	NA	103 (21.2)	383 (78.8)	11 (5.6)	184 (94.4)
Is soap present at the HWF?	NA	NA	47 (45.6)	56 (54.4)	–	–
After using toilet	391 (80)	95 (20)	471 (97)	15 (3)	89 (55)	74 (45)
After cleaning baby’s bottom	44 (9)	442 (91)	478 (98)	8 (2)	107 (64)	61 (36)
Before cooking	114 (24)	354 (76)	45 (9)	441 (91)	59 (35)	111 (65)
Before eating	466 (96)	20 (4)	454 (93)	32 (7)	129 (73)	47 (27)
Before feeding baby	44 (10)	442 (90)	41 (8)	445 (92)	91 (58)	65 (42)
Before suckling baby	21 (4)	464 (96)	–	–	8 (8)	90 (92)
Food hygiene (*N* = 486)					*N* = 195	
Food cooked thoroughly	262 (54)	224 (46)	269 (55)	217 (45)	–	–
Serving utensils washed thoroughly with soap	242 (49)	244 (51)	243 (50)	243 (50)	78 (40)	115 (60)
Cooked food stored safely using tight lid	132 (27)	354 (73)	132 (27)	354 (73)	61 (58)	44 (42)
Leftovers reheated thoroughly	86 (18)	400 (82)	49 (10)	431 (89)	–	–
Food protected from insects or flies	133 (27)	353 (73)	172 (35)	309 (66)	46 (77)	14 (23)
Cooked food stored at appropriate temperature	73 (15)	413 (75)	53 (11)	428 (88)	54 (66)	28 (34)
Kitchen cloths clean	50 (10)	436 (90)	31 (6)	450 (93)	134 (83)	28 (17)
Kitchen cleaned	194 (40)	292 (60)	175 (36)	306 (63)	–	–
Uncooked food separated from cooked food	9 (2)	477 (98)	16 (3)	465 (96)	46 (38)	75 (62)
Drinking water treatment (*N* = 486)					*N* = 195	
Boiling	354 (82)	132 (18)	184 (37.9)	302 (62.1)	–	–
Chlorination	284 (66)	202 (34)	17 (3.5)	469 (96.5)	–	–
Cloth filter	164 (38)	322 (62)	12 (2.5)	474 (97.5)	–	–
Decantation	5 (1)	481 (99)	1 (0.2)	485 (99.8)	–	–
Solar disinfection	2 (0.5)	484 (99.5)	1 (0.2)	484 (99.8)	–	–
Water filter	3 (0.6)	483 (99.4)	5 (1)	481 (99)	–	–
Storage water containers condition					*N* = 187	
Clean	–	–	353 (73)	131 (27)	140 (75)	47 (25)
Storage container covered	–	–	405 (83.3)	78 (16)	153 (82)	33 (18)
Storage container lid clean	–	–	325 (72)	80 (17)	133 (78)	37 (22)
Hygiene condition of the toilets (*N* = 470)					*N* = 181	
Whether toilet was clean	–	–	313 (67)	154 (33)	109 (60)	72 (40)
Whether toilet floor/walls were clean	–	–	299 (64)	167 (34)	109 (60)	72 (40)
Whether flies were visibly present	–	–	142 (30)	325 (69)	119 (66)	61 (34)
Whether there was no stench (bad smell)	–	–	205 (44)	261 (56)	111 (62)	69 (38)
Child feces disposal (*N* = 486)						
Burying	32 (7)	454 (93)	5 (1)	288 (59)		–
Throwing in the bush	5 (1)	481 (99)	2 (0.7)	291 (98.3)	–	–
Throwing in the latrine	339 (70)	147 (30)	250 (55)	43 (9)	–	–
Throwing in the garbage pit	3 (1)	483 (99)	11 (2.3)	282 (58)	–	–
Throwing in the river	6 (1)	480 (99)	7 (1.4)	286 (59)	–	–

### Handwashing with soap at critical moments.

Handwashing with soap was found to be poor in both urban and rural areas. During the FGDs, most of the respondents said that factors influencing low-handwashing practices are the cost of the soap and lack of awareness on the benefits of handwashing practices. Besides, in rural areas, respondents said that limited water availability prevent them from handwashing in the critical moments. One respondent said, “*water is a very big problem around…we walk two kilometres to fetch water…once water is brought home…. It must be used carefully, rarely by washing hands.*”

The respondents were asked to report both their knowledge and practice of key hygiene behaviors and then observed (through structured observations) to assess whether they actually practice the behaviors (Table 4). Although 97% reported washing their hands after using the toilet, the structured observation showed that only 46% washed their hands after using toilet. Similarly, 66% were observed washing their hands before eating compared with a reported 93%. Also, 85.8% of respondents reported having handwashing stations at their homes, whereas only 21.2% of the households surveyed had handwashing stations. Of these households with handwashing stations, 54.4% of them had soap available (Table 4). Table 5 shows the chi-square analysis of variables associated with the presence of handwashing stations in the household. The presence of a handwashing station within the household was associated with household’s water source/accessibility (χ^2^ = 16.3 and *P* = 0.003). Households connected directly to the town’s piped water system were more likely to have handwashing stations (handwashing sink with running water from the tap) than households not connected to the piped water systems. In addition, there was an association between the households having a handwashing station and the education level (χ^2^ = 11 and *P* = 0.005) of the respondents and their household monthly income (χ^2^ = 16 and *P* = 0.005).

**Table 5:** Variables associated with presence of handwashing facilities at households

		Handwashing station present in household house?				
		No		Yes		
		*n*	*f* (%)	*n*	*f* (%)	
Head of the household	Male-headed household	342	89.3	41	10.7	0.72 (χ^2^ = 3.24)
	Female-headed household	98	95.1	5	4.9	
Education level	No formal education	43	93.5	3	6.5	0.005 (χ^2^ = 10.49)
	Primary school education	265	91.7	24	8.3	
	Secondary school and above	47	78.3	13	21.7	
Marital status	Single	43	89.6	5	10.4	0.276 (χ^2^ = 3.9)
	Married/cohabiting	321	89.7	37	10.3	
	Divorced/separated	33	100	0	–	
	Widow	43	91.5	4	8.5	
Wealth quantile	High	308	89	38	11	0.278 (χ2 = 2.56)
	Second/third	6	100	0	–	
	Fourth	43	95.6	2	4.4	
Residence ownership	Privately owned house	375	90.8	38	9.2	0.892 (χ^2^ = 0.23)
	Relative house	17	89.5	2	10.5	
	Rental house	48	88.9	6	11.1	
Source of water	Borehole	36	97.3	1	2.7	0.003 (χ^2^ = 16.3)
	Buy from vendors	31	93.9	2	6.1	
	Fetch from a river/canal/spring	45	91.8	4	8.2	
	Fetch from other sources	188	94.5	11	5.5	
	Connected to the water supply	140	83.3	28	16.7	
Family size	1–4	156	90.2	17	9.8	0.705 (χ^2^ = 0.698)
	5–8	250	91.2	24	8.8	
	9 and above	440	87.2	5	12.8	
Income (USD)	49 and below	162	94.2	10	5.8	0.005 (χ^2^ = 15.866)
	50–99	104	89.7	12	10.3	
	100–149	43	89.6	5	10.4	
	150–199	15	78.9	4	21.1	
	200 and above	31	75.6	10	24.4	

### Water-related behaviors.

Thirty-five percent of the households reported being connected to the town’s piped water system, whereas 40.9% indicated collecting water from other sources (e.g., public tap water, and rain), and the remaining collect water from boreholes, a river/canal/spring, or vendors. The distance from the household to the water source was between 10 and 400 m. When respondents were asked to mention different known water-treating methods, the most common methods mentioned were boiling (82%) followed by chlorination (66%). However, when asked if they treat water before drinking, 55% of respondents reported not treating their water, although the rest reported treating their drinking water through various methods including boiling (37%), filtration using a piece of cloth (3%), and chlorination (3%) (Table 4). Findings from FGDs showed that treatment of water at household level was performed only when water is collected from unsafe sources including rivers and lakes. One of the respondents said, “*People boil water when it is from unsafe sources or when water is from the valleys and rivers.*” Majority believed that water from piped water systems or springs is safe and clean, therefore, do not need any treatment. One of the respondent stated “*In the past we were boiling water but because Babati Water and Sanitation Authority (BAWASA) had not supplied us water but nowadays we are connected and drink directly from taps without any treatment.*” “*We believe that, water from springs is not contaminated and we have been drinking it for centuries without getting sick.*”

### Sanitation-related behaviors including disposal of child feces.

Forty percent of the available toilets in the study area were not cleaned and had a bad smell and were home to flies. More than half of surveyed households use water and traditional brooms to clean their toilets. General cleaning in the household including toilets was done every morning. Flush/pour flush toilets were cleaned using powder/liquid soap, disinfectants, and toilet brushes. Furthermore, 70% of respondents had knowledge on proper child feces disposal, whereas only 50% were observed disposing children feces in the toilet (Table 4).

### Food hygiene.

Fifteen percent of respondents were familiar with the key food hygiene practices. Respondents described food hygiene as cooking food thoroughly (54%), washing serving utensils thoroughly with soap (50%), storing cooked foods safely using tight lids (27%), making the kitchen clean (40%), and reheating leftovers thoroughly (18%). However, only 2% of respondents were aware of the need to separate cooked foods from uncooked foods. Again, 55% of the households reported cooking foods thoroughly, 27% reported storing cooked food with tight lids, although only 10% reported reheating food. There were variations on reported and observed practices as described in Table 4. For example, 50% of respondents reported washing kitchen utensils with soap, which is contrary to observed practice of 40%.

### Menstrual hygiene behavior.

The majority of girls (72%) reported using pieces of clothes, with few using sanitary pads. During FGD sessions, one of the respondents living in the outskirts of the town said, “*Our fellows living in town use cottons/sanitary pads, you know buying cotton/sanitary pads sometimes you can’t afford but pieces of clothes to us, is affordable.*” Keeping sanitary pads/clothes on for more than 2 hours was a common practice. Putting used disposable pads in school bags and later mixing them with domestic garbage were reported as a normal practice among school girls. It was further reported that it was not always possible to properly dry clothes/sanitary pads before they are reused and drying underwear and sanitary items under beds/mattress was a common practice. “*I am using pieces of clothes, when they get dirty I wash and dry them under my bed, after completing menstruation cycle I keep them, for the following month*”, this was reported by one of the school girl during FGD session. There were no changing rooms in schools, instead common latrines were used, and this was a challenge to school girls during menstruation. In some schools (12%), emergency pads were provided to girls, but only one piece was provided per day. The common challenge for menstrual hygiene in many schools was the absence of a place to dispose sanitary pads. The common practice is to dispose them in toilets, causing them to clog. In this regard, in many schools where flush toilets were installed, girls said that they preferred pit latrines rather than the flush ones. Girls reported reoccurrence of diseases such as vaginal yeast infections and urinary tract infections which were believed to occur as a result of poor hygiene, particularly menstrual hygiene practices especially for those using sanitary pads. “*It is true that these sanitary pads have effects if not properly used….…..Let’s speak the truth; there are some of my daughters who have been using them and yet experience fungal infections which myself I had never suffered from, so it’s better to use pieces of cloth.*” Five percent of school girls declared missing school/classes during menstruation for some reasons including lack of sanitary materials, illnesses associated with menstruation, lack of freedom for playing with friends or boys, fear of shame in case the bleeding become uncontrolled in front of people, and lack of private place/changing room for girls. One of the student from one school said, “*Yes, sometimes I miss school as I fear perhaps, I may wet myself because a piece of cloth may not help me much, but if I get sanitary pads I will be more safe and comfortable while in class and I am sure my skirt will not get wet even if my bleed is heavy.*”

### Hygiene practices of customers using public toilets.

On average, 1,053 customers daily used the toilets located at the bus station. Although there was no running water connection into the toilets, soap and water (kept in a bucket) were available at the two handwashing stations. Customers pay 200 Tanzanian shillings (TZS, ≈0.1 USD) to use the facility. Throughout the observation, 24% of the customers using the bus station toilets in Babati town washed their hands after using the toilet (Table 6). The main reported reasons for not washing hands included a long queue for the handwashing station and limited time available for customers who were in transit. Handwashing practice was more common among women (38%) than men (18%) (*P* = 0.04). A significant proportion of customers who washed their hands after using the toilet did not use soap (32%) (Table 6).

**Table 6:** Handwashing practices among customers attending public toilets

Variable	Female		Male	
	*n*	%	*n*	%
Ward name				
Babati	365	35	265	11
Bagara	676	65	2,241	89
Place				
Market	327	31	101	4
Bus stand	714	69	2,405	96
Handwashing after using toilet				
Yes	397	38	460	18
No	644	62	2,046	82
Materials used to wash hands after toilet use (among those who washed hands)				
Water only	84	21	49	11
Soap and water	311	77*	409	89*
Total	1,041	100	2,506	100

* Percentage of customers who washed their hands using water and soap out of those observed washing hands after using toilet.

### Sanitation conditions in public spaces.

At the Babati town’s bus station, there are eight pour flush toilets (five for women and three for men) and four urinals. Our spot-check observation showed that the floor, walls, pans, and urinals of the toilet were moderately clean, with flies and a moderately bad smell. The toilet was cleaned regularly by attendants. Cleaning materials including soap and disinfectants were also available in the toilets, although the attendants did not have protective gears for cleaning except coats. The main town market has five flush toilets (three for women and two for men). Unlike the bus station toilets, all the market toilet cubicles had a bad smell with a lot of flies, floors were wet, and running water was not available in the toilets. In a few cubicles, feces were observed in the pans, and cleaning materials were not available (Supplemental Table 3). Cash collections of 200 TZS (≈0.1 USD) from every customer who used the toilet were mainly reported to be used for maintenance and cleaning.

### Sanitation and hygiene conditions in schools.

In the schools surveyed, more than 29% of the female toilets and 44% of the male toilets were dirty (presence of human excreta and/or worms could be seen on the floor/drop holes or on the walls). In addition, 41% of the female toilets and 47% of the male toilets had a very strong smell. Teachers’ toilets were in a better condition in terms of cleanliness than toilets for students. Among the 34 teachers’ toilets spot-checked, 8.8% were found to be dirty (Supplemental Table 3). During FGDs, most of the boy respondents admitted practicing open defecation while they are at school and girls reported that they would hold stool or urine until they could go home. One of the respondents said, “*Our toilets are very dirty and during break time you will find a long queue there, so it’s better to go to the bushes behind the school buildings for short call/defecation rather than going to the school toilets.*” Some of the students reported that their parents have forbidden them to drink water while they are in school so that they would not use the school toilets. WASH facilities in schools were not accessible to students with special needs. None of the schools visited had accessible sanitation and hygiene facilities for students with physical impairments. Even in the schools organized for students with disabilities, the sanitation and hygiene facilities were not accessible to this particular group.

In the majority of school settings, there was just one tap located outside the toilets and shared by the whole school for washing hands. In some schools, tippy taps (*vibuyu chirizi* in Kiswahili) were commonly used because they are cheap to construct compared with other handwashing stations. Most boy respondents admitted not washing their hands when going for a short call but rather before getting their meals believing that they do not contaminate their hands when they go for a short call. However, girls reported washing their hands after using the toilet and before eating. In all cases, soap was rarely used. Regarding water treatment, most students reported that they drink directly from the taps. The situation is different in private schools where water is treated through boiling and sometimes using water guard. Most students preferred tap water that they claimed having better taste.

## DISCUSSION

Babati Town Council, like many other urban areas in Tanzania and the developing world, has a rapidly growing population. However, the growth in urban populations is not happening hand in hand with increased demand for improved sanitation and hygiene services provision. Challenges related to sanitation have been reported to exist in several developing countries’ small towns.^1^ This study clearly highlights that Babati town depends entirely on on-site sanitation systems characterized mainly by “dry” toilets, an important factor that needs to be considered for future planning for the town’s sanitation services. Town planning is, however, an issue with many peri-urban and urban areas growing in an unplanned manner, posing great challenges to current and future provision of sanitation and hygiene services.

The survey findings from this study revealed that sanitation and hygiene behaviors of the individuals and the community in the study area are influenced mainly by sociodemographic, economic, and cultural factors. These include source/accessibility of water in the household/institution, education level, residence ownership, and wealth quantile.

The type of water source/accessibility in the household/institution had significant influence on the type of latrine owned by that particular household/institution. People whose households are not connected to the water pipe systems may find it difficult and unpractical to construct and use flush/pour flush toilets (“wet” sanitation type). As a result, they will opt for other types of toilets, particularly pit latrines. The findings from this study are similar to findings from the study conducted in India which revealed an association between the type of latrine owned by a household/institution and water accessibility in the household/institution.^12^

The study revealed further that the type of latrine owned by the household was strongly associated with the wealth quantile level of the household. Households which belong to high wealth quantile are likely to have improved latrines when compared with households which belong to low wealth quantiles. Wealthy families are good economically and able to afford modern houses with piped water systems and flush toilets when compared with poor families which may not afford modern infrastructures. The findings from this survey are mirrored by findings from the study conducted in rural communities of developing countries which reported an association between the household wealthy quintile level and latrine type.^13^

The study revealed further that the level of education of the household head had a strong association with the type of latrine the household owns. It is likely that educated people are likely to have good knowledge on the health benefits of having good sanitation/improved latrines when compared with uneducated ones. These findings are in line with findings from the study conducted in Ethiopia which revealed a strong association between the latrine type, utilization, and social characteristics/status of the household individuals.^14^

The household survey data revealed further that the level of income and sanitation facility was insignificant which is contrary to other studies that showed a very strong association between participants’ level of income and the type of latrine ownership.^1,15^ However, low income as a barrier toward installation of improved sanitation facility emerged in all the FGDs. It is often too expensive for households with low income to afford having a flush/pour flush toilet, as they have to ensure water connection systems are in place as well as costs for constructing wet sanitation facilities. Most households in Babati town, similar to other growing towns/cities, are of low income and, therefore, unable to incur costs to construct flush/pour flush toilets and get connected to the piped water systems which are paid on a monthly basis. The findings from FGDs are consistent with a study conducted in Bangladesh which reported a strong association between the type of toilets owned by the household and good economic status.^16^ Future planning for the town’s sanitation services and full sanitation chain will need to consider these findings in selecting an appropriate solution.

For hygiene, the presence of handwashing stations in the household was strongly associated with the education level of the household head, source/accessibility of water, and level of income of the household head. The reason for this could be due to the fact that educated people could be aware of the health benefits of having handwashing stations in their homes for people to wash their hands. Again, they are likely to have good income as majority could be employed and salaried and likely to afford piped water systems in their homes. The level of education and access of water in the house could influence installation of handwashing stations probably in the kitchens and toilets for people to wash hands. These findings are in line with findings from study carried out in Kenya.^17^

As expected, the reported practices on handwashing during the four critical moments differed significantly from the observed practices. Handwashing with soap has been linked to a 30–48% reduction in the risk of endemic diarrhea,^18–20^ but its compliance during critical moments is quite low in the study area posing risk for individuals. In fact, improved hygiene (handwashing) and sanitation (toilets) have more impact than drinking water quality on health outcomes, specifically reductions in diarrhea, parasitic infections, morbidity and mortality, and increases in child growth.^8^ That is to say, most of endemic diarrhea is not waterborne, but rather transmitted from person to person by poor hygiene practices.

Most of the households with children younger than 5 years in the study area cooked food twice a day and fed children multiple times throughout the day. Only around one-third (58%) of the households stored food in a container with a tight lid and maintained a safe temperature. There is evidence to suggest that bacteria can grow rapidly in food that is poorly stored or stored for more than 4–6 hours.^21^ It is not surprising, therefore, that eating cold leftovers is associated with a heightened risk of diarrhea. The cleanliness of serving utensils before serving food and thorough reheating leftover food before feeding/eating behavior are also suboptimal in the study area. Previous studies suggest that food hygiene behaviors are underpinned by physical (kitchen surface and access to cleaning products), social norms around using cold leftovers, and biological settings (animal accessing kitchen and presence of flies) in the households.^22^ Initiatives to improve food hygiene behaviors in such environments should consider changes in the physical, social, and biological settings, most importantly the kitchen settings although it would be challenging considering other key behavioral determinants including motivational drivers.^23^

Disposal of child feces in the toilets was not practiced by the majority, believing that it is harmless, and some were disposed in the gardens or garbage pits in the household surroundings. This could be due to lack of awareness on the health risks associated with poor disposal of child feces. Studies have estimated that the unsanitary disposal of child feces may result in a 63% increase in diarrhea.^24^ Poor child feces disposal practices have also been found to be associated with a heightened risk of undernutrition, intestinal worms, environmental enteropathy, and death.^25,26^ Child feces are often poorly disposed of because toilets are not designed for, or indeed used by, small children. Findings from the current study are similar to findings from a study carried out among semi-pastoral communities in the northern Tanzania.^27,28^

Menstrual hygiene management was seen to be a neglected issue in the study area, and little attention has been given on this subject. The reason could be due to sociocultural and economic issues including lack of awareness on the health consequences of poor menstrual hygiene and financial constrains the women in the study area have. The findings from this study are in line with studies carried out elsewhere where women were unable to practice good menstrual hygiene because of financial and cultural challenges.^29–31^ For women of reproductive age to enjoy a dignified life, be healthy, and productive, it is essential that they are able to manage menstruation effectively, although several issues have been identified in the area under study which compromise these aspects. Addressing menstrual hygiene challenges in the area under study requires access to appropriate water, sanitation, and hygiene services, including clean water for washing clothes used to absorb menstrual blood and having a place to dry them, having ladies’ private changing rooms, facilities to dispose used clothes and pads, and access to information to understand the menstrual cycle and how to manage menstruation hygienically and access to information to men to also support.

Study Limitation: This study did not focus on solid waste management aspects, and therefore, replication of the findings should take this into consideration.

## CONCLUSION

Babati town still has a low level of safely managed sanitation services and low compliance on good hygiene behaviors, despite the current level of ongoing national effort to improve the sanitation condition and hygiene behaviors. There is an urgent need to ensure that the sanitation and hygiene services and behaviors along the value chain are improved. This would involve both hygiene behavior change promotion being integrated into WASH programs and planning safely managed sanitation services for Babati town and other growing towns with similar settings.

## Supplemental tables

Supplemental materials
