# Supplementary material for: Sanitation and Hygiene Practices in Small Towns in Tanzania: The Case of Babati District, Manyara Region
Source: Am J Trop Med Hyg. 2020 Aug 17;103(4):1726–34. doi: 10.4269/ajtmh.19-0551 (PMC7543826; doi:10.4269/ajtmh.19-0551)
Supplement: Supplementary file 1 [file tpmd190551.SD1.doc]

**Supplementary Materials**

**Supplementary Table1. Socio-demographic characteristics of study participants**

| **Socio-demographic characteristics** | **N** | **%** |
| --- | --- | --- |
| **Respondent gender (n=486)** | | |
| Female | 305 | 63 |
| Male | 181 | 37 |
| Single | 48 | 9.9 |
| Married‎‎/Cohabiting | 358 | 73.7 |
| Divorced‎/Separated | 33 | 6.8 |
| Widow | 47 | 9.7 |
| **Education level (n = 395)*** |  |  |
| No formal education | 46 | 11.6 |
| Primary education | 289 | 73.2 |
| Secondary education and above | 60 | 15.2 |
| **Family size (n = 486)** |  |  |
| 1 to 4 | 173 | 35.6 |
| 4 to 8 | 274 | 56.4 |
| 9 and above | 39 | 8 |
| **Head of household (n = 486)** | | |
| Male Headed Household | 383 | 78.8 |
| Female Headed Household | 103 | 21.2 |
| **Primary occupation of head of household (n = 486)** | | |
| Farmer (crop grower) | 277 | 57 |
| Businessperson | 82 | 17 |
| Salaried worker | 39 | 8 |
| Casual laborer | 24 | 5 |
| Fishermen | 13 | 3 |
| Livestock Keeper | 11 | 2 |
| Others | 40 | 10 |
| **Household ownership (n = 486)** | | |
| Privately owned house | 413 | 85 |
| Relative house | 19 | 43.9 |
| Rental house | 54 | 11.1 |
| **Wealth quintile (n = 397)** | | |
| First | 346 | 87.2 |
| Second‎‎/Third‎/Fourth | 51 | 12.8 |
| Fifth Quintile | 0 | 0 |
| **Household Income (US$ per months)** |  |  |
| 49 and below | 172 | 43.4 |
| 50 – 99 | 116 | 29.3 |
| 100 – 149 | 48 | 12.1 |
| 150 – 199 | 19 | 4.8 |
| 200 and above | 41 | 10.4 |
| Average household income | 101 USD | |
| Minimum monthly income | 04 USD | |
| Maximum monthly income | 1,434 USD | |

**The level of education data was collected later after being left out of the original questionnaire. Due to this situation, some of the*

*respondents were not available.*

**Supplementary Table 2. Toilet ownership and types of sanitation facilities in Babati Town**

|  | **Sanitation status** | **n** | **%** |
| --- | --- | --- | --- |
| Ownership of a toilet (n=486) | Private toilet | 438 | 90.1 |
|  | Shared toilet | 32 | 6.6 |
| Types of toilets owned by a household (n=470) | No facility | 16 | 3.3 |
|  | Flush/pour flush directly in an open space | 18 | 3.8 |
|  | Flush or pour-flush to septic tank or pit latrine | 137 | 29.1 |
|  | Pit latrine without slab/open pit | 93 | 19.8 |
|  | Pit latrine with slab | 202 | 43 |
|  | VIP latrine | 20 | 4.3 |
| Materials of superstructure of latrine (n=470) | Cement/burnt bricks | 335 | 71.3 |
|  | Clay | 24 | 5.1 |
|  | Wood | 55 | 11.7 |
| Type of the latrine roofing materials (n=470) | Grass/Stalks | 39 | 8.3 |
|  | Aluminum sheets | 10 | 2.1 |
|  | Cloths/polypropylene/plastics | 7 | 1.5 |
|  | Aluminum sheets/tiles | 260 | 55.3 |
| Type of the floor/slab (n=470) | Poor aluminum sheets/cracks | 48 | 10.2 |
|  | Grasses | 23 | 4.9 |
|  | No roof | 139 | 29.6 |
|  | Concrete slab | 318 | 67.7 |
|  |  |  |  |
|  | Mud slab | 148 | 31.5 |
|  | Wood | 4 | 0.9 |

**Supplementary Table 3. Hygiene condition of toilets in schools and colleges**

| Description |  | (n) | Percentage (%) |  |
| --- | --- | --- | --- | --- |
|  |  |  |  |  |
| Cleanliness – Floor/walls - Male toilet | Dirty (Presence of human excreta on the floor or drop hole / wet | 15 | 44.1 |  |
|  | slab/worms can be seen/dirty walls) |  |  |  |
|  |  |  |  |
|  |  |  |  |  |
|  | Mildly dirty (dirty but not like above) | 10 | 29.4 |  |
|  | Clean (Absence human excreta on the floor or drop hole / slab is dry) | 9 | 26.5 |  |
|  |  |  |  |  |
| Cleanliness – Floor/walls - Female toilet | Dirty (Presence of human excreta on the floor or drop hole / wet | 10 | 29.4 |  |
|  | slab/worms can be seen/dirty walls) |  |  |  |
|  | Mildly dirty (dirty but not like above) | 13 | 38.2 |  |
|  | Clean (Absence human excreta on the floor or drop hole / slab is dry) | 11 | 32.4 |  |
|  |  |  |  |  |
| Cleanliness – Floor/walls - Teacher's toilet | Dirty (Presence of human excreta on the floor or drop hole / wet | 3 | 8.8 |  |
|  | slab/worms can be seen/dirty walls) |  |  |  |
|  |  |  |  |  |
|  | Mildly dirty (dirty but not like above) | 12 | 35.3 |  |
|  |  |  |  |  |
|  | Clean (Absence human excreta on the floor or drop-hole / slab is dry) | 19 | 55.9 |  |
|  |  |  |  |  |
| Smell - male toilet | Very strong bad smell (chocking) | 16 | 47.1 |  |
|  |  |  |  |  |
|  | Moderately bad smell | 14 | 41.2 |  |
|  |  |  |  |  |
|  | No smell | 4 | 11.8 |  |
|  |  |  |  |  |
|  | Artificial/flavored smell/ fragrances | 0 | 0.0 |  |
|  |  |  |  |  |

| Smell - female toilet | Very strong bad smell | 14 | 41.2 |
| --- | --- | --- | --- |
|  |  |  |  |
|  | Moderately bad smell | 13 | 38.2 |
|  |  |  |  |
|  | No smell | 7 | 20.6 |
|  |  |  |  |
|  | Artificial/flavored smell/ fragrances | 0 | 0.0 |
|  |  |  |  |
| Smell - teachers' toilet | Very strong smell | 2 | 5.9 |
|  | Moderately bad smell | 9 | 26.5 |
|  |  |  |  |
